# Correlation of Digital Rectal Examination and Anorectal Manometry with Patient-Reported Outcomes Among Women with Fecal Incontinence

**DOI:** 10.1007/s00192-024-05848-7

**Published:** 2024-08-20

**Authors:** Alayne Markland, Mary Ackenbom, Uduak Andy, Ben Carper, Eric Jelovsek, Douglas Luchristt, Shawn Menefee, Rebecca Rogers, Vivian Sung, Donna Mazloomdoost, Maria Gantz, NICHD Pelvic Floor Disorders Network

**Affiliations:** 1 Department of Medicine, University of Alabama at Birmingham, Geriatric Research, Education, and Clinical Center (GRECC) Birmingham VA Medical Center, 11G, 700 South 19th Street, Birmingham, Alabama 35233, USA; 2 Department of Obstetrics, Gynecology and Reproductive Sciences, University of Pittsburgh/Magee Womens Research Institute, Pittsburgh, PA, USA; 3 Department of Obstetrics and Gynecology, Hospital of University of Pennsylvania, Philadelphia, PA, USA; 4 Biostatistics and Epidemiology Division, RTI International, Research Triangle Park, NC, USA; 5 Department of Obstetrics and Gynecology, Duke University Medical Center, Durham, NC, USA; 6 Department of Obstetrics and Gynecology, Kaiser Permanente San Diego, San Diego, CA, USA; 7 Department of Obstetrics and Gynecology, Albany Medical College, Albany, NY, USA; 8 Department of Obstetrics and Gynecology, Warren Alpert Medical School of Brown University, Women’s & Infants Hospital, Providence, RI, USA; 9 Eunice Kennedy Shriver National Institute of Child Health and Human Development, Bethesda, MD, USA

**Keywords:** Anorectal manometry, Anorectal physiology, Clinical examination, Fecal incontinence, Patient-reported outcome measures, Women

## Abstract

**Introduction and Hypothesis:**

Standardized digital rectal examination (DRE) correlates with anorectal manometry (ARM) measures. However, less is known about the relationship between DRE/ARM measures and patient-reported outcomes (PROs), especially among women with fecal incontinence (FI). Our aims were to evaluate associations between DRE and ARM measures and compare PROs with diagnostic evaluation measures for women with FI.

**Methods:**

We analyzed data from the parent clinical trial, Controlling Anal incontinence by Performing Anal exercises with Biofeedback or Loperamide (CAPABLe). We pooled data from randomized women who completed standardized ARM, DRE, and validated PROs at baseline and 12 and 24 weeks post-treatment initiation. PROs included FI severity, impact on quality of life, and bowel diary data. We analyzed ARM pressure and volume data and DRE using the Digital Rectal Examination Scoring System (DRESS) resting and squeeze mean scores. We used Spearman Rank Correlation to measure associations between the ARM measures and mean DRESS scores, and between PROs and ARM/DRESS scores.

**Results:**

Among 291 randomized women with ARM and DRE data, the correlation between DRESS and ARM resting measures was 0.196 (*p*<0.001) and between squeeze measures was 0.247 (*p*<0.001). At most timepoints, PROs more consistently correlated with squeeze ARM pressures and squeeze DRESS scores than resting measures.

**Conclusions:**

We found weak correlations between ARM and DRE measures and between those measures and PROs. Although DRE and ARM are commonly used diagnostic measures among women with FI, the weak correlations with patient-reported symptoms raises questions about their utility in clinical care.

## Introduction

Fecal incontinence (FI), the involuntary loss of solid or liquid feces, is also referred to accidental bowel leakage. In a systematic review of 38 studies across several countries, the prevalence was 7.7% across all ages, with a range of 2–21% based on FI frequency, and similar prevalence rates for men and women [1]. Frequent or severe FI can have a devastating impact on the lives of those affected, leading to social withdrawal and depression and contributing to the decision to go into a nursing home [2].

Clinical guidelines and consensus statements for FI evaluation and treatment do not specify the timing of performing anorectal manometry (ARM) for the evaluation of FI in women to guide therapy [3–5]. Expert opinion suggests that ARM might not be necessary to initiate treatments aimed at improving stool consistency with medications or introducing behavioral interventions, such as anal muscle exercises and biofeedback. Often, ARM evaluation is utilized when first-line treatment options are insufficient in improving symptoms and quality of life or when surgical intervention may be considered.

To further evaluate the utility of ARM among women seeking first-line treatment options, we conducted a planned secondary analysis of a large trial (Controlling Anal incontinence by Performing Anal Exercises with Biofeedback or Loperamide, CAPABLe) where women with FI were randomized to nonsurgical therapies in a 2×2 factorial design [6]. The objective of this analysis was to evaluate correlations between digital rectal examination (DRE) and ARM measures and evaluate correlations between DRE/ARM measures and PROs at baseline and at 12 and 24 weeks after treatment initiation. Based on existing evidence on ARM and PROs, we hypothesized that ARM measures would have stronger correlations with PROs than DRE findings.

## Materials and Methods

This study was a planned secondary investigation of a multi-site randomized controlled trial of treatment for women with FI conducted by the Pelvic Floor Disorders Network (PFDN) [6]. The methods and results of the randomized trial have been previously published [6, 7]. In brief, women were randomized in a 2×2 factorial design using a 0.5:1:1:1 allocation to one of four groups:

1. Oral placebo plus education only
2. Placebo and anal sphincter exercise training using ARM-assisted biofeedback
3. Oral loperamide plus education only
4. Loperamide and anal sphincter exercise training using ARM-assisted feedback

Education consisted of a publicly available pamphlet from the National Institute of Diabetes and Digestive and Kidney Diseases (NIDDK) with deletion of a single reference to the drug loperamide. Study procedures for all sites were approved by site Institutional Review Boards and all participants provided written informed consent.

The study included women who were ≥ 18 years old who had had FI at least monthly over the past 3 months that was bothersome enough to desire treatment. Participants completed the clinical examination, including the DRE and the ARM, as well as PROs at baseline (prior to randomization), and at 12 and 24 weeks after treatment initiation.

All sites had trained clinical professionals performing the DRE and the ARM measures according to a standardized protocol [8]. During a centralized training process using compensated volunteers, we certified the skills necessary to perform a standardized DRE assessment called the Digital Rectal Examination Scoring System (DRESS) [9]. The DRESS has two components: a resting score (range 0 to 5) and a squeeze score (range 0 to 5). Higher scores represent high DRE resting tone and very strong voluntary squeeze ability. Resting scores are discerned as 0=“no discernable tone,” 1=“very low tone,” 2=“mildly decreased tone,” 3=“normal,” 4=“elevated tone, snug,” and 5=“very high tone, a tight anal canal.” Squeeze scores are determined as 0=“no discernable increase in tone with squeezing effort,” 1=“slight increase,” 2=“fair increase but below normal,” 3=“normal,” 4=“strong squeeze,” and 5=“very strong squeeze.” The DRESS has been previously demonstrated to correlate with ARM resting and squeeze pressures [9]. Trained clinicians completed the DRESS at baseline and at 12 and 24 weeks. We compared average resting and squeeze DRESS scores with ARM measures and PROs.

Trained clinicians also conducted ARM measures at baseline, 12 weeks, and 24 weeks using mcompass (Medspira, Minneapolis, MN, USA) equipment. The mcompass system used a tablet computer that connected wirelessly to an air-charged catheter. Resting and squeeze anal sphincter pressures (mmHg) were ascertained with the catheter at 0-, 1-, and 2-cm depths to define the maximum resting and squeeze pressures at the high-pressure zone (HPZ). To test sensation to balloon filling, the clinicians recorded the volume (ml) of air at the first sensation of rectal distention. Comparative data with high-resolution manometry equipment have been reported [10].

Patient-reported outcomes (PROs) for FI severity, impact on quality of life, and bowel diaries were completed at baseline and at 12 and 24 weeks. The St. Mark’s (Vaizey) score measured FI severity, along with the Pelvic Floor Disorders Distress Inventory (PFDI) subscale Colo-Rectal Anal Distress Inventory (CRADI) [11, 12]. The Colorectal Anal Impact Questionnaire (CRAIQ) subscale from the Pelvic Floor Impact Questionnaire (PFIQ) and the impact subscale from the Modified Manchester Health Questionnaire (MMHQ) measured the impact of FI on quality of life [12, 13]. Bowel diary data collected over 7 days included the number of accident-free days, the mean number of leaks per day, and the number of pad changes per day for FI.

After summarizing standard descriptive statistics of the women with baseline DRE and ARM data (*n*=291), we used Spearman Rank Correlation to measure the strength of the associations between the ARM measures and mean DRESS scores, as well as the associations between the ARM/DRESS scores and the PROs at baseline and at 12 and 24 weeks. Correlations with a *p* value of <0.05 were considered statistically significant.

## Results

From this multi-site clinical trial, a total of 291 women completed baseline clinical examination with the DRE, the ARM evaluation, and PRO measures. The demographic characteristics of the 291 study participants are shown in Table 1. Over one third of participants (36%) were between the ages of 60 and 69 years, nearly three quarters (74%) were white, non-Hispanic, and nearly all (96%) had English as a primary language. More than half reported having private insurance and/or Medicaid/Medicare.

Digital rectal examination-ascertained DRESS scores correlated with ARM pressures for resting measures at baseline (Table 2; 0.196, *p*<0.001) and for squeeze measures (0.247, *p*<0.001). Resting DRESS scores also correlated with squeeze ARM pressures (0.220, *p*<0.001). However, squeeze DRESS scores did not correlate with resting ARM pressures (0.053, *p*=0.37).

Across all timepoints (Table 3), PROs correlated with ARM and DRE findings, but this was related almost exclusively to squeeze parameters. The St. Mark’s (Vaizey) score inversely correlated with higher squeeze ARM pressures at baseline (−0.201, *p*<0.001), 12 weeks (−0.169, *p*=0.008), and 24 weeks (−0.175, *p*=0.006). The St. Mark’s (Vaizey) score also correlated inversely with higher squeeze DRESS scores at 24 weeks (−0.191, *p*=0.002), but not at baseline or 12 weeks. The CRADI score correlated inversely with higher squeeze DRESS scores at 12 weeks (−0.206, *p*=0.001) and 24 weeks (−0.251, *p*<0.001), but not at baseline. CRADI scores correlated inversely with higher squeeze ARM measures at 12 weeks only (−0.179, *p*=0.006). The CRADI score at 12 weeks was the only PRO that correlated inversely with rest DRESS scores (−0.186, *p*=0.004).

We found consistent correlations between impact on quality life PROs and squeeze DRE and ARM findings at baseline, but only DRE findings remained significantly correlated at 24 weeks (Table 3). Specifically, CRAIQ score inversely correlated with squeeze ARM pressures at baseline only (−0.145, *p*=0.01), whereas CRAIQ scores correlated inversely with squeeze DRESS scores at baseline (−0.133, *p*=0.02) and 24 weeks (−0.154, *p*=0.01). We found similar inverse correlations with MMHQ impact subscale scores and squeeze ARM pressures at baseline (−0.129, *p*=0.03). Similar to CRAIQ scores, MMHQ scores correlated inversely with squeeze DRESS scores at both baseline (−0.127, *p*=0.03) and 24 weeks (−0.260, *p*<0.001).

From the bowel diary PROs, the average pad changes per day had the most consistent correlations with ARM and DRE findings across most timepoints. We found inverse correlations between average pad changes per day and higher squeeze ARM pressures at baseline (−0.135, *p*=0.02), 12 weeks (−0.135, *p*=0.03), and 24 weeks (−0.137, *p*=0.03), as well as higher squeeze DRESS scores at baseline (−0.122, *p*=0.03) and at 24 weeks (−0.192, *p*=0.002). Pad usage correlated inversely with resting ARM pressures (−0.133, *p*=0.02) only at baseline. We found less consistent findings for accident-free days and average leaks per day across other ARM and DRE findings. Leaks per day correlated with volume of first air sensation on ARM at baseline (0.120, *p*=0.04), 12 weeks (0.137, *p*=0.03), and at 24 weeks (0.168, *p*=0.009) and correlated inversely with squeeze ARM pressures at 12 weeks only (−0.160, *p*=0.01).

Accident-free days correlated inversely with volume of first air sensation on ARM at 12 weeks (−0.157, *p*=0.01) and at 24 weeks (−0.182, *p*=0.004) and correlated with squeeze ARM pressures at 12 weeks only (0.157, *p*=0.01).

## Discussion

Overall, we found weak correlations between PROs and standardized DRE and ARM measures among women with FI. Fecal incontinence and colorectal symptom severity measures across timepoints correlated more consistently with squeeze ARM and DRE findings than other PROs, such as impact on quality of life and bowel diary measures. We found inconsistent correlations with PROs and resting DRE and ARM findings. Our findings question the need to perform more invasive assessments, such as ARM, as they may not accurately represent symptom severity over time in women participating in a FI treatment clinical trial that utilized first-line treatments.

Very few studies of patients with FI reported correlations between DRE findings and ARM measures, with existing data suggesting correlations among all types of measures, including PROs [9, 14–18]. Some studies have reported stronger correlations between DRE findings and ARM (coefficients of 0.82 for resting pressure and 0.81 for squeeze pressure) [9]; however, our findings indicated weaker coefficients for resting pressure (0.196) and for squeeze pressure (0.247) between DRE findings and ARM measures. This may have resulted from differences in eligibility criteria for FI symptoms for the parent clinical trial (women only), the presence of pelvic floor dysfunction, and our wider variety of clinicians performing the evaluation across multiple sites, as compared with the initial validation study using an existing clinical database at a single institution, including men and women [7, 9]. We found very few other studies that utilized a validated DRE scoring system when describing differences between DRE findings and ARM measures [15].

For studies that compared ARM and PRO measures to predict treatment among patients with FI, one recent study of 276 men and women found that PROs for FI symptom severity and impact on quality of life, along with only one ARM measure (5-s endurance squeeze increment pressure), predicted the need for surgical intervention [17]. Another study reached a similar verdict that ARM may not be needed in all FI patients; however, the authors concluded that endoanal ultrasound may be necessary to further evaluate anal sphincter deficits [15]. In a contrasting study to ours from a colorectal surgery cohort, resting ARM pressures correlated with PRO measures for FI severity; whereas squeeze pressure did not correlate with PRO measures for FI severity. In this same study, the authors concluded that ARM measures were not good at predicting the extent of anal sphincter deficits as evaluation with endoanal ultrasound [14]. Other studies that have evaluated predictors for FI nonsurgical and surgical interventions did not analyze ARM measures [19, 20]. Performing ARM may or may not impact treatment response and more data are needed to inform clinical care.

The main strength of this study was the planned secondary analysis of a large, multi-center, randomized controlled clinical trial with a well-characterized study population [6]. Additional strengths include the fact that subjective assessment of FI symptoms and impact were performed using validated PROs and objective evaluation using a validated DRE scoring system and standardized measures across sites with uniform training and certified clinicians across three separate timepoints [8]. Mean number of FI episodes at baseline was 1.6 leaks per day, providing a significantly impacted population to characterize correlations between measures. Finally, a broad range of PROs were included in this analysis, which is important, given the lack of an understanding of well-defined predictors for FI treatment response [21, 22].

Nonetheless, most of the current study population was white and post-menopausal, limiting the generalizability to other populations. ARM measures and PROs for this study were difficult to compare with other studies owing to the differences in the measures performed. A recent consensus statement discusses the need for better standardization for ARM measures, regardless of type of ARM equipment used [23]. Last, we did not perform endoanal ultrasound evaluation or defecography for this clinical trial and our ability to compare these additional evaluations of anorectal structure and function are limited.

With increased focus on PROs and a weak correlation between diagnostic testing measures, future studies should further evaluate the utility of DRE findings compared with ARM measures, as well as PROs, for predicting treatment outcomes for nonsurgical and surgical FI treatments. This study provides evidence to inform expectations of diagnostic evaluation testing for nonsurgical treatment options and did not directly compare ARM findings with DRE findings in predicting PROs or predicting changes or response with treatment. Additional research should focus on factors that can be readily obtained at an office visit and that do not require more invasive clinical evaluation. For women with FI, the higher cost and discomfort associated with invasive testing with ARM and endoanal ultrasound may not be warranted. This information may allow providers to prescribe nonsurgical treatments for FI symptoms without needing additional ARM evaluation.

## Figures and Tables

**Table 1:** Baseline demographic characteristics and clinical examination characteristics of study participants

Characteristics	All subjects (*N*=291)

Demographic, *n* (%)	
Age (years)	
< 40	7 (2)
40–49	28 (10)
50–59	67 (23)
60–69	105 (36)
70–79	67 (23)
80+	17 (6)
Race/ethnicity	
White, non-Hispanic	216 (74)
Black, non-Hispanic	42 (14)
Hispanic	26 (9)
Other/multiple races	7 (2)
Primary language	
English	278 (96)
Other/unknown	13 (4)
Insurance	
Private insurance	176 (60)
Medicaid/Medicare	154 (53)
Clinical examination, mean (SD)	
ARM measures	
Maximal resting pressure (mmHg)	39.9 (17.4)
Maximal squeeze pressure (mmHg)	73.0 (33.2)
Volume of air at first sensation (ml)	26.3 (17.8)
DRE measures	
DRESS score—resting	2.0 (0.9)
DRESS score—squeeze	1.8 (1.1)
PROs	
FI severity	
St. Mark’s/Vaizey score (0–24)	14.2 (4.1)
CRADI score (0–100)	50.3 (21.9)
Impact on quality of life	
MMHQ impact (0–100)	63.1 (25.9)
CRAIQ (0–100)	43.5 (28.5)
Bowel diary measures	
Accident-free days	2.9 (2.2)
Average leaks per day	1.6 (1.8)
Average pad-change per day	0.6 (1.0)

*ARM* anorectal manometry, *DRE* digital rectal examination, *PRO* patient-reported outcome, *FI* fecal incontinence, *DRESS* Digital Rectal Examination Scoring System, *CRADI* Colorectal Anal Distress Inventory, *MMHQ* Modified Manchester Health Questionnaire, *CRAIQ* Colorectal Anal Impact Questionnaire

**Table 2:** Correlations of resting and squeeze digital rectal examination measures with anorectal manometry measures at baseline

	ARM measure					
	Maximum resting pressure of the anal canal at the HPZ (mm of Hg)			Maximum squeeze pressure of the anal canal at the HPZ (mm of Hg)		
DRE measure	*N*	Spearman correlation	*p* value	*N*	Spearman correlation	*p* value

DRESS score—resting (mean, range 0 to 5)	287	0.196	<0.001	291	0.220	<0.001
DRESS score—squeeze (mean, range 0 to 5)	287	0.053	0.371	291	0.247	<0.001

Positive correlations indicate that as DRESS scores increase ARM pressures also increase

*ARM* anorectal manometry, *DRE* digital rectal examination, *PRO* patient-reported outcome, *HPZ* high-pressure zone, *DRESS* Digital Rectal Examination Scoring System

**Table 3:** Correlations of digital rectal examination measures and anorectal manometry measures to patient-reported outcomes at baseline and 12 and 24 weeks after treatment initiation

PRO	ARM measures									DRE measures					
	Maximum resting pressure (mm of Hg)			Maximum squeeze pressure (mm of Hg)			Volume of air at first sensation (mi)			DRESS score—resting (mean, range Oto 5)			DRESS score—squeeze (mean, range Oto 5)		
	*N*	Spearman correlation	*p* value	*N*	Spearman correlation	*p* value	*N*	Spearman correlation	*p* value	*N*	Spearman correlation	*p* value	*N*	Spearman correlation	*p* value

FI and colorectal symptom severity measures (range)															
St. Mark’s/Vaizey score (0–24, increased score represents more severe symptoms)^a^															
Baseline	287	−0.065	0.28	291	−0.201	<0.001	289	0.025	0.67	291	−0.038	0.51	291	−0.050	0.39
12 weeks	238	−0.051	0.43	242	−0.169	0.008	243	0.126	0.05	247	−0.079	0.21	247	−0.088	0.16
24 weeks	243	−0.102	0.11	243	−0.175	0.006	246	0.050	0.43	254	−0.073	0.24	254	−0.191	0.002
CRADI scores. (0–100, increased score represents more severe symptoms)^a^															
Baseline	287	−0.022	0.71	291	−0.081	0.17	289	0.075	0.20	291	−0.110	0.06	291	−0.091	0.12
12 weeks	227	0.011	0.86	230	−0.179	0.006	231	0.122	0.06	234	−0.186	0.004	234	−0.206	0.001
24 weeks	225	0.012	0.86	225	−0.126	0.05	228	0.060	0.36	236	0.003	0.96	236	−0.251	<0.001
Impact on quality of life measures (range)															
CRAIQ score (0–100, increased score represents greater impact)^a^															
Baseline	287	−0.026	0.67	291	−0.145	0.01	289	0.070	0.23	291	−0.020	0.73	291	−0.133	0.02
12 weeks	224	0.092	0.16	227	−0.089	0.18	228	0.116	0.07	231	−0.127	0.05	231	−0.108	0.10
24 weeks	223	0.015	0.82	222	−0.086	0.20	225	0.067	0.32	233	−0.049	0.45	233	−0.154	0.01
MMHQ Impact Score (0–100, increased score represents greater impact)^a^															
Baseline	285	−0.010	0.87	289	−0.129	0.03	289	0.000	0.99	289	−0.092	0.11	289	−0.127	0.03
12 weeks	221	0.050	0.46	224	−0.118	0.07	225	0.082	0.22	228	−0.086	0.19	228	−0.095	0.15
24 weeks	218	0.046	0.49	218	−0.050	0.46	220	0.088	0.19	228	−0.041	0.53	228	−0.260	<0.001
Bowel diary measures															
Accident-free days ^b^															
Baseline	284	0.041	0.50	288	0.039	0.50	286	−0.074	0.21	288	−0.063	0.28	288	0.059	0.32
12 weeks	234	0.042	0.52	238	0.157	0.01	238	−0.157	0.01	241	0.032	0.61	241	0.032	0.62
24 weeks	235	0.034	0.59	235	0.045	0.49	238	−0.182	0.004	246	0.022	0.72	246	0.094	0.14
Average leaks per day ^a^															
Baseline	284	−0.081	0.18	288	−0.031	0.60	286	0.120	0.04	288	0.065	0.26	288	−0.042	0.47
12 weeks	234	−0.056	0.39	238	−0.160	0.01	238	0.137	0.03	241	−0.013	0.84	241	−0.051	0.43
24 weeks	235	−0.008	0.90	235	−0.044	0.50	238	0.168	0.009	246	−0.009	0.88	246	−0.091	0.15
Average pad-change per day^a^															
Baseline	284	−0.133	0.02	288	−0.135	0.02	286	0.029	0.63	288	0.003	0.95	288	−0.122	0.03
12 weeks	234	−0.003	0.96	238	−0.135	0.03	238	0.106	0.10	241	−0.043	0.51	241	−0.099	0.12
24 weeks	235	−0.069	0.29	235	−0.137	0.03	238	0.101	0.12	246	−0.075	0.24	246	−0.192	0.002

*ARM* anorectal manometry, *DRE* digital rectal examination, *PRO* patient-reported outcome, *DRESS* Digital Rectal Examination Scoring System, *CRADI* Colorectal Anal Distress Inventory, *MMHQ* Modified Manchester Health Questionnaire, *CRAIQ* Colorectal Anal Impact Questionnaire

a Inverse (negative) correlations with the ARM and DRE measures represent a lower symptom burden, a lower impact on quality of life, a decreased number of leaks per day, and decreased pad changes per day with increases in ARM and DRE measures

b Positive correlations with the ARM and DRE measures represent higher numbers of accident-free days with increases in ARM and DRE measures

## Data Availability

Data accessible through the Pelvic Floor Disorders Network website: pelvicfloordisorders.org/AboutUs/PublicData.
